# Barriers, Solutions, and Opportunities for Adapting Critical Care Clinical Trials in the COVID-19 Pandemic

**DOI:** 10.1001/jamanetworkopen.2024.20458

**Published:** 2024-07-12

**Authors:** Deborah Cook, Shipra Taneja, Karla Krewulak, Nicole Zytaruk, Kusum Menon, Rob Fowler, François Lamontagne, Michelle E. Kho, Bram Rochwerg, Marie-Hélène Masse, François Lauzier, Katie O’Hearn, Neill K. J. Adhikari, Karen E. A. Burns, Karen J. Bosma, Shane English, Dayre McNally, Alexis F. Turgeon, Laurent Brochard, Melissa Parker, Lucy Clayton, Asgar Rishu, Angie Tuttle, Nick Daneman, Dean Fergusson, Lauralyn McIntyre, Laurel Kelly, Sherrie Orr, Peggy Austin, Sorcha Mulligan, Kirsten Fiest

**Affiliations:** 1 Departments of Medicine, Health Research Evidence & Impact, McMaster University, Hamilton, Canada; 2 Department of Health Research Evidence & Impact, McMaster University, Hamilton, Canada; 3 Department of Critical Care, University of Calgary, Calgary, Alberta, Canada; 4 Department of Pediatrics, University of Ottawa, Ottawa, Canada; 5 Interdepartmental Division of Critical Care, University of Toronto, Toronto, Canada; 6 Department of Critical Care, Université de Sherbrooke, Sherbrooke, Canada; 7 School of Rehabilitation Science, McMaster University, Hamilton, Canada; 8 Centre de recherche du Centre Hospitalier Universitaire de Sherbrooke, Sherbrooke, Canada; 9 Department of Anesthesiology and Critical Care Medicine, Division of Critical Care Medicine, Université Laval, Québec City, Canada; 10 CHU de Québec-Université Laval Research Center, Québec City, Canada; 11 Children’s Hospital of Eastern Ontario Research Institute, Ottawa, Canada; 12 Keenan Research Center, Li Ka Shing Knowledge Institute, Unity Health Toronto, Toronto, Ontario; 13 Department of Medicine, Division of Critical Care Medicine, Schulich School of Medicine & Dentistry, University of Western Ontario, London, Canada; 14 Department of Medicine (Critical Care), University of Ottawa, Ottawa, Canada and Clinical Epidemiology Program, Ottawa Hospital Research Institute, Ottawa, Canada; 15 Department of Pediatrics, University of Ottawa, Ottawa, Canada; 16 Departments of Pediatrics and Emergency Medicine, McMaster University, Hamilton, Canada; 17 Ottawa Hospital Research Institute, Ottawa, Canada; 18 Division of Infectious Diseases, Department of Medicine, Sunnybrook Health Sciences Centre, University of Toronto, Toronto, Canada; 19 Ottawa Hospital Research Institute, University of Ottawa, Ottawa, Canada; 20 Department of Critical Care, University of Ottawa, Ottawa, Canada; 21 St Joseph’s Healthcare, Hamilton, Canada; 22 Department of Pediatrics, McMaster University, Hamilton, Canada; 23 Applied Health Research Centre, Li Ka Shing Knowledge Institute, Unity Health Toronto, St Michael’s Hospital, Toronto, Ontario, Canada

## Abstract

**Importance:**

The COVID-19 pandemic created unprecedented challenges for clinical trials worldwide, threatening premature closure and trial integrity. Every phase of research operations was affected, often requiring modifications to protocol design and implementation.

**Objectives:**

To identify the barriers, solutions, and opportunities associated with continuing critical care trials that were interrupted during the pandemic, and to generate suggestions for future trials.

**Design, Setting, and Participants:**

This mixed-methods study performed an explanatory sequential analysis involving a self-administered electronic survey and focus groups of principal investigators (PIs) and project coordinators (PCs) conducting adult and pediatric individual-patient randomized trials of the Canadian Critical Care Trials Group during the COVID-19 pandemic. Eligible trials were actively enrolling patients on March 11, 2020. Data were analyzed between September 2023 and January 2024.

**Main Outcomes and Measures:**

Importance ratings of barriers to trial conduct and completion, solutions employed, opportunities arising, and suggested strategies for future trials. Quantitative data examining barriers were analyzed using descriptive statistics. Data addressing solutions, opportunities, and suggestions were analyzed by qualitative content analysis. Integration involved triangulation of data sources and perspectives about 13 trials, synthesized by an interprofessional team incorporating reflexivity and member-checking.

**Results:**

A total of 13 trials run by 29 PIs and PCs (100% participation rate) were included. The highest-rated barriers (on a 5-point scale) to ongoing conduct during the pandemic were decisions to pause all clinical research (mean [SD] score, 4.7 [0.8]), focus on COVID-19 studies (mean [SD] score, 4.6 [0.8]), and restricted family presence in hospitals (mean [SD] score, 4.1 [0.8]). Suggestions to enable trial progress and completion included providing scientific leadership, implementing technology for communication and data management, facilitating the informed consent process, adapting the protocol as necessary, fostering site engagement, initiating new sites, streamlining ethics and contract review, and designing nested studies. The pandemic necessitated new funding opportunities to sustain trial enrollment. It increased public awareness of critical illness and the importance of randomized trial evidence.

**Conclusions and Relevance:**

While underscoring the vital role of research in society and drawing the scientific community together with a common purpose, the pandemic signaled the need for innovation to ensure the rigor and completion of ongoing trials. Lessons learned to optimize research procedures will help to ensure a vibrant clinical trials enterprise in the future.

## Key Points

**Question:**

What barriers, solutions, and opportunities were associated with continuing critical care randomized trials during the COVID-19 pandemic, and which strategies could help to optimize trials in the future?

**Findings:**

In this mixed-methods study using responses from study investigators of 13 clinical trials, trial principal investigators and coordinators identified serious challenges, including decisions to pause all clinical research, focus on COVID-19 studies, and restricted family presence in hospital. Suggestions to ensure trial progress and completion highlighted the importance of scientific leadership, leveraging technology, facilitating informed consent, protocol adaptation, site initiation and engagement, timely document approval, and nested studies.

**Meaning:**

These results suggest that the COVID-19 pandemic catalyzed innovations to advance clinical trials that are relevant beyond the pandemic.

## Teaser

This mixed-methods study of clinical trials conducted during the COVID-19 pandemic surveys principal investigators and project coordinators and summarizes challenges, opportunities, and suggestions for running trials in a pandemic setting.

## Introduction

Randomized trials that were under way before the COVID-19 pandemic experienced numerous challenges to continuation and completion. A prepandemic systematic review of 172 trials found that the most common reasons for trial discontinuation were overestimated prevalence of the target condition, overestimated interest in the trial intervention, new external evidence, administrative or participant burden, and insufficient funding.^1^ The pandemic posed additional challenges impacting every phase of research operations, threatening premature closure and trial integrity, often requiring modifications to protocol design or implementation.^2,3^

The National Institutes of Health Center for Advancing Translational Science aimed to convert the research trauma of the pandemic into what it described as “post-traumatic growth,” with the goal of making the translational research enterprise stronger, more resilient, and more effective.^4^ Aligned with this concept, we hypothesized that strategies developed in response to the COVID-19 pandemic by investigations in the intensive care unit (ICU) may have broader application. The objectives of this qualitative study were to identify the barriers, solutions, and opportunities associated with conducting critical care trials during the pandemic, and to generate suggestions for future trials.

## Methods

We conducted an explanatory sequential mixed-methods study involving a self-administered survey followed by virtual focus groups. Data were collected from January 2023 to January 2024. Reporting methods align with guidelines for qualitative^5^ and mixed-methods research.^6,7^ This study received approval by the Hamilton Research Ethics Board. A priori informed consent for survey and focus group participation was digitally documented by each participant via email using an embedded institutional REDCap (REDCap Consortium) link. This study followed the Standards for Reporting Qualitative Research (SRQR) reporting guideline.

### Participants

Using the Canadian Critical Care Trials Group (CCCTG) database as the purposive sampling frame, we invited principal investigators (PIs) and project coordinators (PCs) of all adult and pediatric individual-patient parallel-group randomized trials affiliated with the consortium that were actively recruiting patients before March 11, 2020. We excluded trials launched after March 11, 2020, and alternate trial designs (eg, cluster, umbrella, basket, and platform trials). Subsequently, the same individuals were invited to participate in a focus group as rationalized below.

### Design

#### Survey

We designed this survey following rigorous methods.^8^ Initial items were generated during virtual and in-person meetings of the study team, including 25 trialists and project coordinators. Domains of interest were characteristics of the trials and participants, barriers to trial completion, solutions for trial completion, new opportunities arising, and suggestions for future trials. Survey items addressed diverse topics such as ethics, legal and regulatory issues, protocol modifications, operational adaptations, and financial and human resources. Pretesting feedback by the research team helped to revise the instrument, and 2 PIs and 2 PCs of noneligible CCCTG trials commented on the flow, salience, and acceptability of the items,^8^ identified any unusual phrases, and recorded survey completion time. Feedback was used to streamline the instrument and separate compound questions.

We invited 4 PIs and 4 PCs of noneligible studies to judge the clinical sensibility of the instrument using a 5-point Likert scale (5 representing positive valence). Mean (SD) scores were 4.8 (0.4) for construct validity, 4.8 (0.4) for face validity, 4.3 (0.7) for clarity, 4.8 (0.5) for redundancy, and 3.9 (1.4) for content validity. The latter views about missing items prompted additions and reinforced the focus group plans.

Both closed and open-ended response options were used for survey formats. Closed formats were binary, nominal, and ordinal (barrier scores were reported as 1 [irrelevant] to 5 [very important]). An email invitation to potential PI and PC survey participants was sent with an individualized link via REDCap (eAppendix 1 in Supplement 1). Two email reminders were sent 2 weeks apart, as necessary. Incentives were not offered.

#### Focus Groups

We conducted focus groups using qualitative descriptive methodology to invite participants to clarify and elaborate on solutions, opportunities, and suggestions identified in the survey.^9,10^ Email invitations were sent to all PIs and PCs. One of 3 qualitative researchers facilitated the focus groups (K.K., S.T., K.F.) via videoconferencing (Zoom); a second recorded field notes.

The interview guide was developed based on survey results that required additional details and insights (eAppendix 2 in Supplement 2). Focus groups lasted approximately 1 hour, held separately for PIs and PCs. Preliminary survey results were shared. Participants were asked to share perspectives from their own trial experiences and observations. Focus groups were recorded, transcribed verbatim, and anonymized. To provide feedback and to affirm, adjust, or amplify results, we integrated member-checking throughout the analysis, sharing preliminary findings from the survey and initial focus groups in subsequent focus groups.^11^

### Statistical Analysis

#### Quantitative Data

Quantitative survey data only focused on barriers and were closed-ended, analyzed using descriptive statistics. Binary data are presented as counts and percentages. Ordinal data are presented as means (with SDs).

We compared barrier ratings from PI vs PC respondents and adult vs pediatric-based respondents using Student *t* tests. Quantitative data analysis was conducted using Excel version 16.78.3 (Microsoft).

#### Qualitative Data

First, open-ended survey data related to solutions, opportunities, and suggestions for future trials were reviewed by 4 investigators (S.T., K.K., D.J.C., K.F.) using conventional content analysis to classify concepts.^10^ Open coding of the data was initially performed independently in duplicate (K.K., S.T.), followed by meetings to compare, consolidate, and refine the categories. Using the revised codebook, a second round of focused coding was conducted (S.T., K.K.), then resultant categories and concepts were modified or affirmed in larger group discussion (K.K., S.T., D.J.C., K.F.); these were used to develop the interview guide.

Second, focus group findings solutions, opportunities, and suggestions underwent a qualitative descriptive analysis.^9^ We analyzed data using an inductive approach to conventional content analysis, whereby codes were derived directly from the data^10^ to create a descriptive summary of findings with minimal interpretive inference. Analytic work was documented using an audit trail. Saturation was reached after 4 of 6 focus groups. NVivo version 14 (QSR International) was used for data management and coding.

#### Mixed-Methods Integration

Integration of the survey and focus group data occurred at multiple stages.^7^ The survey clinical sensibility results and survey findings informed the interview guide, justified the need for qualitative data, and highlighted areas for intentional exploration. Triangulation was achieved by the interprofessional research team synthesizing outcomes from both perspectives in both datasets.

## Results

### Trials

All 13 eligible trials were included (100% participation rate); 10 enrolled critically ill adults and 3 enrolled critically ill children (Table 1). Trial interventions included mechanical ventilation,^15,19^ cycle ergometry,^14^ medications (antibiotic duration,^13^ stress ulcer prophylaxis,^20^ hormonal therapy,^22,24^ vitamin C^18^), blood transfusion strategies,^17,21^ fluid resuscitation,^16,23^ and family engagement.^12^

**Table 1.** Characteristics of Randomized Trials

Trial	Trial name	ClinicalTrials.gov identifier	Population (target sample size)	Intervention	Primary outcome
Fiest et al^12^	ACTIVATE (Activating Family Caregivers in the Identification, Prevention, and Management of Delirium)	NCT04099472	Pediatric patients and adult family member dyads (198, revised to 64)	Family administered delirium prevention, detection, and management vs standard care	Change in proportion of family members with anxiety
Daneman et al^13^	BALANCE (Bacteremia Antibiotic Length Actually Needed for Clinical Effectiveness)	NCT03005145	Adult (3626)	7 vs 14 d of antibiotics	90-d mortality
Kho et al^14^	CYCLE (Critical Care Cycling to Improve Lower Extremity Strength)	NCT03471247; NCT02377830 (internal pilot)	Adult (360)	In-bed cycling and routine physiotherapy activities vs routine physiotherapy activities alone	PFIT-s at 3 d after ICU discharge
Burns et al^15^	FAST-NAWC (Frequency of Screening and SBT Technique Trial–North American Weaning Collaboration)	NCT02969226	Adult (760)	Screening frequency (once daily vs at least twice daily) and Spontaneous Breathing Test technique (pressure support vs T-piece)	Time-to-successful extubation
Rochwerg et al^16^	FISSH (Fluids in Septic Shock)	NCT03677102	Adult (1096)	Fluid resuscitation with normal saline vs Ringer’s lactate	30-d mortality
Turgeon et al^17^	HEMOTION (Hemoglobin Transfusion Threshold in Traumatic Brain Injury Optimization)	NCT03260478	Adult (742)	Liberal red blood cell transfusion threshold (100 g/L) vs restrictive red blood cell transfusion threshold (70 g/L)	Glasgow Outcome Scale-extended at 6 mos
Masse et al^18^	LOVIT (Lessening Organ Dysfunction with Vitamin C)	NCT03680274	Adult (800)	Intravenous high-dose vitamin C vs placebo	Mortality or persistent organ dysfunction at 28 d
Bosma et al^19^	PROMIZING (Proportional Assist Ventilation for Minimizing the Duration of Mechanical Ventilation)	NCT02447692	Adult (558)	Proportional assist ventilation vs pressure support ventilation	Time-to-successful liberation from invasive mechanical ventilation in days
Deane et al^20^	REVISE (Re-Evaluating the Inhibition of Stress Erosions)	NCT03374800	Adult (4800)	Intravenous pantoprazole vs placebo	Clinically important upper gastrointestinal bleeding within 90 d
English et al^21^	SAHaRA (Aneurysmal Subarachnoid Hemorrhage: Red Blood Cell Transfusion And Outcome)	NCT03309579	Adult (740)	Liberal red blood cell transfusion threshold (100 g/L) vs restrictive red blood cell transfusion threshold (80 g/L)	Modified Rankin score at 12 mos
Stress Hydrocortisone In Pediatric Septic Shock^22^	SHIPSS (Stress Hydrocortisone in Pediatric Septic Shock)	NCT03401398	Pediatric (1032, revised to 500)	Intravenous hydrocortisone vs placebo	New or progressive multiorgan dysfunction syndrome at 28 d
Parker et al^23^	SQUEEZE (Septic Shock Reversal is Quicker in Pediatric Patients Randomized to an Early Goal Directed Fluid-Sparing Strategy vs Usual Care)	NCT03080038	Pediatric (400)	Early goal-directed fluid sparing strategy vs usual care	Difference (in hours) in time-to-shock reversal
Rapid Normalization of Vitamin D Deficiency in PICU^24^	VITdALIZE-KIDS (Vitamin D: Rapid Normalization in Critically Ill Children)	NCT03742505	Pediatric (766)	Enteral vitamin D vs placebo	Health related quality of life, including mortality at 28 d

Abbreviations: ICU, intensive care unit; PFIT-s, Physical Function Test for ICU-score.

**Figure 1.**
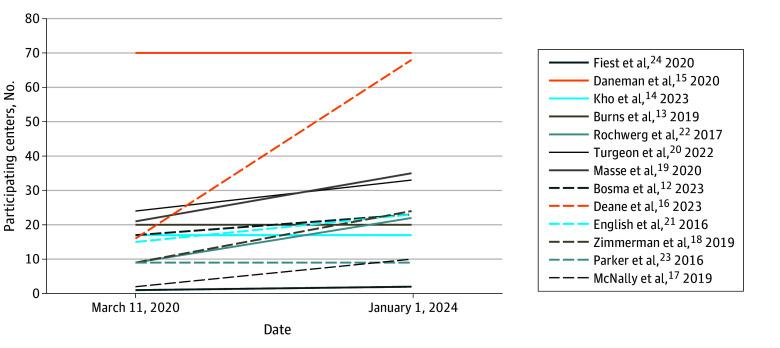
Number of Participating Centers

**Figure 2.**
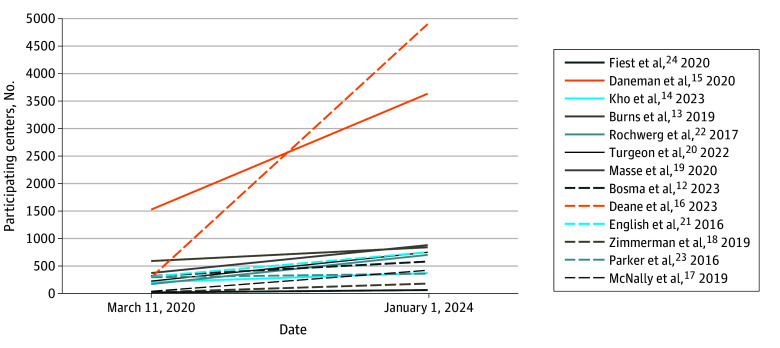
Number of Participants Enrolled

When the pandemic was declared on March 11, 2020, trials were at different stages of completion. From then until January 1, 2024, additional participating sites were initiated (Figure 1), and many patients were enrolled (Figure 2). Together, 11 research networks enrolled patients at 194 centers in 15 countries (Table 2). Nine trials (69.2%) have completed recruitment, and 4 have been published.^25,26,27,28^

**Table 2.** Funding, Collaborators, and Trial Status

Trial	Trial name	Major peer-review funding	Participating countries	Collaborating consortia	Status
Fiest et al^12^	ACTIVATE (Activating Family Caregivers in the Identification, Prevention, and Management of Delirium)	CIHR	Canada	CCCTG, CCIRNet	Complete
Daneman et al^13^	BALANCE (Bacteremia Antibiotic Length Actually Needed for Clinical Effectiveness)	CIHR, NHMRC, MRCNZ	Australia, Canada, Israel, New Zealand, Saudi Arabia, Switzerland, US	CCCTG, ANZICS-CTG, AMMI CRN, CCIRNet	Complete
Kho et al^14^	CYCLE (Critical Care Cycling to Improve Lower Extremity Strength)	CIHR, ACT, AMOSO	Canada, US, Australia	CCCTG, CCIRNet	Published^25^
Burns et al^15^	FAST-NAWC (Frequency of Screening and SBT Technique Trial–North American Weaning Collaboration)	CIHR, PSI	Canada, US	CCCTG, CCIRNet	Complete
Rochwerg et al^16^	FISSH (Fluids in Septic Shock)	CIHR, PSI	Canada, Saudi Arabia	CCCTG, CCIRNet	Enrolling
Turgeon et al^17^	HEMOTION (Hemoglobin Transfusion Threshold in Traumatic Brain Injury Optimization)	CIHR, ACT	Canada, UK, France, Brazil	CCCTG, CTRC, UKCCRG, PACT	Published^26^
Masse et al^18^	LOVIT (Lessening Organ Dysfunction with Vitamin C)	Lotte and John Hecht Memorial Foundation	Canada, France, New Zealand	CCCTG	Published^27^
Bosma et al^19^	PROMIZING (Proportional Assist Ventilation for Minimizing the Duration of Mechanical Ventilation)	CIHR-Industry, CIHR	Canada, France, Saudi Arabia, Italy, Spain, Greece, Argentina	CCCTG, REVA, CCIRNet	Complete
Deane et al^20^	REVISE (Re-Evaluating the Inhibition of Stress Erosions)	CIHR, NHMRC, ACT, PSI	Canada, Australia, Saudi Arabia, UK, US, Kuwait, Pakistan, Brazil	CCCTG, ANZICS-CTG, Sepsis Canada, CCIRNet	Published^28^
English et al^21^	SAHaRA (Aneurysmal Subarachnoid Hemorrhage: Red Blood Cell Transfusion And Outcome)	CIHR, MRFF	Canada, Australia, US	CCCTG	Complete
Stress Hydrocortisone In Pediatric Septic Shock^22^	SHIPPS (Stress Hydrocortisone in Pediatric Septic Shock)	CIHR, NICHD, Thrasher Foundation	Canada, US, Brazil, Israel, Saudi Arabia, Singapore, Malaysia, Vietnam, Japan, Pakistan, China	CCCTG, PALISI, PACCMAN, LASI	Enrolling
Parker et al^23^	SQUEEZE (Septic Shock Reversal is Quicker in Pediatric Patients Randomized to an Early Goal Directed Fluid-Sparing Strategy vs Usual Care)	CIHR, CBS	Canada	CCCTG, PERC	Complete
Rapid Normalization of Vitamin D Deficiency in PICU^24^	VITdALIZE-KIDS (Vitamin D: Rapid Normalization in Critically Ill Children)	CIHR	Canada	CCCTG	Enrolling

Abbreviations: AAMI CRN, Association of Medical Microbiology & Infectious Disease Canada Clinical Research Network; ACT, Accelerating Clinical Trials Consortium; AMOSO, Academic Medical Organization of Southwestern Ontario; ANZICS-CTG, Australian and New Zealand Intensive Care Society Clinical Trials Group; CBS, Canadian Blood Services; CCCTG, Canadian Critical Care Trials Group; CCIRNet, Canadian Community ICU Research Network; CIHR, Canadian Institutes of Health Research; CTRC, Canadian Traumatic Brain Injury Research Consortium; LASI, Latin America Sepsis Institute; MRCNZ, Medical Research Council of New Zealand; MRFF, the Australian Medical Research Futures Fund; NHMRC, National Health & Medical Research Council of Australia; NICHD, National Institute of Child Health and Human Development; PACCMAN, Pediatric Acute and Critical Care Medicine Asian Network; PACT, Perioperative Anesthesia Clinical Trials Group; PALISI, Pediatric Acute Lung Injury and Sepsis Investigators; PERC, Pediatric Emergency Research Canada; PSI, Physicians Services Incorporated of Ontario; REVA, Réseau Européen de Recherche en Ventilation Artificielle; UKCCRG, UK Critical Care Research Group.

### Participants

All invited participants engaged in this study (100% response rate), including 18 PIs (3 trials had coprincipal investigators), and 11 PCs (1 managed 2 trials). Of 29 individuals, 17 (58.6%) identified as women and 12 (41.4%) as men.

### Barriers

**Figure 3.**
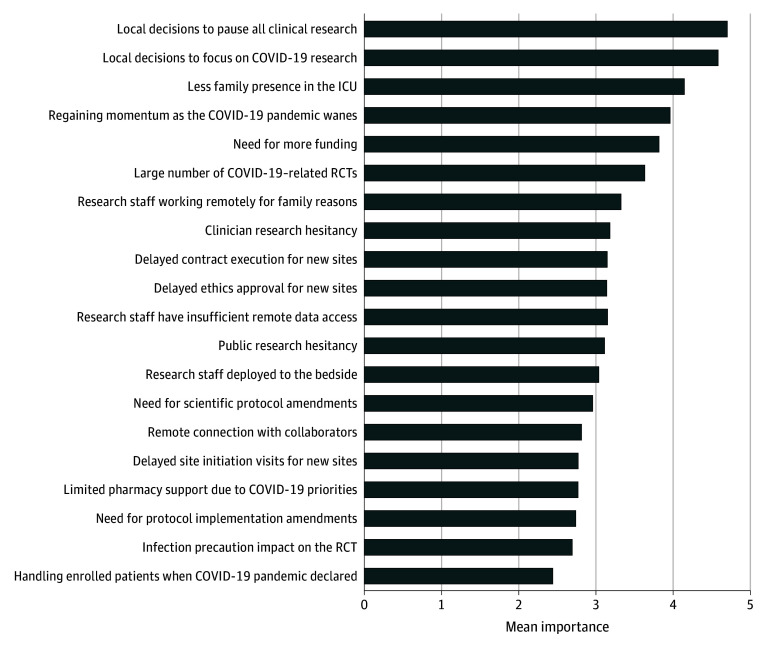
Importance of Barriers to Trial Completion

Participants endorsed many barriers to trial completion (Figure 3). The 3 highest-rated barriers included local decisions to pause all clinical research (mean [SD] score, 4.7 [0.8]), directives to focus on COVID-19–specific studies (mean [SD] score, 4.6 [0.8]), and reduced family presence in the hospital (mean [SD] score, 4.1 [0.8]). The next most important challenges were regaining trial momentum as the pandemic waned and needing additional funding for trial completion. Research delays were highly rated barriers, including delayed ethics approvals and contract execution and deferred new site initiation.

Participants representing trials in adult ICUs rated numerous COVID-19 trials as a greater barrier to completion of their trial than pediatric respondents (mean [SD] barrier score, 4.2 [1.1] vs 1.7 [0.8]; *P* < .001). PCs perceived the deployment of research staff to the bedside during the pandemic to be a larger barrier than PIs (mean [SD] barrier score, 3.7 [1.5] vs 2.4 [1.4]; *P* = .02).

### Solutions Used and Future Suggestions

Participants shared their solutions toward trial progress, categorized as future suggestions. These 8 categories included provide scientific leadership, implement technology for communication and data management, facilitate the informed consent process, adapt the protocol as necessary, foster site engagement, initiate new sites, streamline ethics and contract review, and conduct nested studies.

#### Provide Scientific Leadership

Participants described stepping into diverse leadership roles. “We struck an ICU team that actually talked about all the hospital studies but particularly the ones that would affect the ICU patients,” said a PI participant. “So we had to bring the teams together; it was purposeful…having the studies work together, but also look for opportunities for coenrollment. That was something really positive.” In response to directives to pause all clinical research, stakeholder-informed guidance was also developed on “principles to guide non-COVID–specific research during the pandemic,” as a PC participant described it, highlighting the importance of continuing all types of studies, when safe, feasible, and jurisdictionally admissible.^29^

In response to many new COVID-19 studies, the CCCTG developed a COVID-19 Network of Clinical Trials Networks to improve collaboration among trials across the continuum of care in several settings.^30^ To balance an extreme resetting of research priorities, trial teams also created new processes to transparently monitor and report the composition and progress of the national health research portfolio,^30^ allowing stakeholders to assess whether it reflected Canada’s health priorities.^31^

#### Implement Technology for Communication and Data Management

Methods centers embraced technology-based communication, incorporating virtual site initiation and retraining visits as needed. The pandemic gave license to employ remote data monitoring; investigators’ meetings shifted almost exclusively to videoconferencing. Said a PI, “We adapted quickly.” At enrolling centers, virtual technology improved bedside staff education and departmental coordination across research teams, ensuring sensitive patient and family-centered approaches during the proliferation of COVID-19 studies.^32^

Research staff often worked remotely to screen patients, contact families, and collect data, which continued after on-site presence was permitted. “Screening got a lot more efficient,” said a PI. “You could screen from home in the morning, see if there were eligible participants and then decide whether you needed to come in person that day…and you could screen multiple times in the day.”

#### Facilitate the Informed Consent Process

Participants noted changes to admissible consent encounters. As a PI described it, explicit consent to approach families for research—“which we’re required to do before we can even do the consent process”—was no longer required, so “the teams would…approach directly.” Witnessed telephone consent encounters without immediate signatures were approved. “We had to transition more to… telephone consents because family weren’t around,” said a PC, as “that was obvious…for all trials.” In a pediatric trial, prerandomization vitamin D levels were approved with telephone consent.^24^

Permission for broader consent methods was granted for some trials, expanding “the ways that consent-to-continue could be obtained…by telephone or by e-consent,” said a PC. “So our [ethics board] felt…[with] the shortage of masks…we could use deferred [consent]…and go on.” Admissible consent documentation broadened in some centers, including video recording (eg, Zoom, Teams), faxed, and electronic signatures (eg, REDCap or Docusign). However, concerns were raised about disadvantaging participants with limited technological access or ability, as well as limited health literacy.

#### Adapt the Protocol as Necessary

Participants determined the relevance of their research question to patients with COVID-19, adapting their protocol or its implementation as necessary. To ensure protocol fidelity for a physiotherapist-led cycle ergometry trial, protocol changes were necessary.^33^ Trials captured SARS-CoV-2 status if relevant; some developed a COVID-19 subgroup analysis. A PI reported that an “explicit decision that patients with COVID-19 and on a vasopressor would qualify…as in viral sepsis,” facilitated early completion of 1 trial. The potential immunomodulatory effects of vitamin C for COVID-19^34^ led this team to rapidly launch and complete the harmonized LOVIT-COVID and vitamin C domain of REMAP-CAP trials.^35^

Otherwise, enrollment challenges were substantial. For 1 pediatric sepsis trial, the primary outcome was changed to enhance feasibility; a smaller sample size was required and global collaboration was increased to enhance generalizability.^22^ Four trials^16,18,22,24^ allowed enrollment without biomarker samples—a PC described this as a result of being “flexible…because it was not the main outcome.” For a delirium prevention trial, when bedside survey completion by families was prohibited, the design changed to a pilot trial focused on fewer surveys and the feasibility of home completion.^12^

#### Foster Site Engagement

Fostering site engagement was crucial, particularly where trials were on hold. Tips for negotiating the relaunch of paused trials were shared via periodic site investigator meetings and newsletters. Where recruitment continued, engagement required steady support (eg, clarifying trial eligibility for COVID-19 patients), reduced enrollment targets, and progress reports.

Importantly, participants recognized the burden of the pandemic. “A change we made with the pandemic was recognizing…people’s bandwidth and what was happening at their centers and their ability to do research,” said a PI. “I would say one of the changes we made, intentionally, was slowing down…not pushing as hard.”

#### Initiate New Sites

Participants remarked how large community centers contributed substantially to COVID-19 research.^36,37^ This increased research capacity was extended to help complete enrollment in the non-COVID-19–specific trials in this study. These centers were well-established, with fewer projects to manage, making major contributions. One PC remarked that of “our top five recruiting centers, two of them are coming from the community sites.”

Collaboration with international sites helped with enrollment targets, and in some cases, completion within the original time frame. “This also introduces additional funding opportunities,” said a PC taking a long-term view. “The partnerships we are building with international sites will help us to complete this trial and will also help for finding sites for future trials.”

#### Streamline Ethics and Contract Review

Observing more rapid ethics and contract approval for COVID investigations “than had ever been realized previously,” participants tracked and shared document turnaround times for the non-COVID-19–specific trials. Motivated by the unmasking of previous institutional inefficiencies, under “gentle but persistent pressure” (as described by a PI), ethics review boards in some centers became “way more efficient, so whenever we had modifications, they turned them around fairly quickly” (via a PC participant)*.*

Timely contract execution was experienced by some participants who provided feedback, whereas for others, contract delays remained the norm. “Contracts are usually the really limiting step,” said a PI. “And those were sped up by orders of magnitude for pandemic-related studies, and so there has been some spillover benefit of that, postpandemic.”

#### Conduct Nested Substudies

Several teams performed a substudy or study-within-a-trial (SWAT)—a self-contained study embedded within a trial, to assess alternative ways of delivering, organizing, or measuring a specific trial process.^38^ For example, 1 trial relying heavily on family participation in hospital was halted; an embedded qualitative study was incorporated to understand the experiences of limited family presence at the bedside.

Another team completed a mixed-methods study of patient and family perspectives to inform the definition of a secondary trial outcome of patient-important upper gastrointestinal bleeding.^39^ To explore potentiating effects of acid suppression, a nested COVID-19 cohort substudy using propensity-matching was also designed.^40^

### Opportunities

Participants reported that heightened awareness of the need for critical care research created local funding opportunities by universities, hospitals, and foundations to continue non-COVID-19–specific trials. However, additional peer review funds were crucial for many trials to maintain research staff, regain recruitment momentum, and recoup financial losses.^41^

Trial teams were galvanized “to take the opportunity to innovate—or learn from innovations happening around you,” said a PI*.* Participants appreciated how preexisting platform trials leveraged existing infrastructure^42^ and other platform trials^43,44^ began generating timely evidence. Advances for COVID-19 possible through large-scale collaborations were profiled in the media, underscoring the vital role of science in society. Participants envisioned other opportunities to strengthen the trials ecosystem, given what a PI described as the “increased understanding among health care practitioners and the public about the importance of research and embedding research into clinical care.”

## Discussion

This mixed-methods study analyzed barriers confronted by research teams managing randomized trials during the pandemic while identifying multifaceted mitigating strategies. These included maintaining scientific leadership in crisis and implementing technology for virtual communication and data management. Trial teams suggested facilitating broader consent methods, building flexibility into protocols to ensure feasibility, and adapting them as necessary. Other facilitators included increasing capacity and encouraging accountability of all stakeholders, as participants advertised ethics and contracts turnaround times while remaining sensitive to local circumstances. Embedded substudies were suggested to enrich the scientific foundation of these trials.

The results of this study complement earlier reported pandemic challenges.^45^ A ClinicalTrials.gov registry analysis found significantly more suspended non-COVID-19 trials, without changes to trial completion up to December 2020.^46^ International critical care trialists endorsed that up to February 2021, the main factors slowing clinical studies were local prioritization of COVID-19–specific research, infection control policies, clinical staff workload, and staff safety concerns.^47^ In the second year of the pandemic, decreased activities in many US clinical research centers suggested long-term consequences for trial operations and finances.^48^ In the current study, participants described how the pandemic exacerbated some common trial barriers—more serious for some trials than others—while also producing new challenges. Research teams activated mitigating strategies to continue and complete trials during extenuating circumstances, which are broadly relevant in both pandemic and nonpandemic times.

### Strengths and Limitations

Strengths of this study include clear purpose, staging, and sequencing for the mixed-methods design.^6^ The survey methods were rigorous.^8^ The systematic qualitative methods support the trustworthiness, auditability, credibility, and transferability of the findings.^49,50^ Integration involved triangulation^51^ of data sources (survey and focus groups) and perspectives (PIs and PCs) about trial populations (critically ill adults and children) testing diverse interventions, analyzed by an interprofessional team incorporating reflexivity and member-checking.^52^ All 13 eligible trials were represented.

Limitations of this study include possible respondent recall bias. We calculated the highest-rated barriers to completion overall, not based on pandemic epochs. Strategies suggested should be empirically evaluated, including the effect of digital approaches on equitable research access, environmental impacts, and trial costs.^53^ Some solutions may not apply to unique features of cluster, umbrella, basket or platform trials, or trials unaffiliated with a network. Views from other institutional stakeholders would augment these strategies; collaborative advocacy will help to realize durable gains for the trial landscape.

## Conclusions

In conclusion, the pandemic sparked innovations to ensure the rigor and safety of ongoing trials. While increasing public awareness about the vital role of research in society and drawing the scientific community together with a common purpose, learnings from the pandemic may prove to be useful long thereafter, heralding a stronger, more vibrant clinical trials enterprise in the future.

## Notes

Supplement 1. **eAppendix 1**. Self-Administered Survey Instrument **eAppendix 2.** Focus Group Interview Guide

Supplement 2.
Data Sharing Statement
