# Supplementary material for: Barriers, Solutions, and Opportunities for Adapting Critical Care Clinical Trials in the COVID-19 Pandemic
Source: JAMA Netw Open. 2024 Jul 12;7(7):e2420458. doi: 10.1001/jamanetworkopen.2024.20458 (PMC11245722; doi:10.1001/jamanetworkopen.2024.20458)
Supplement: Supplement 1. — eAppendix 1. Self-Administered Survey Instrument eAppendix 2. Focus Group Interview Guide [file jamanetwopen-e2420458-s001.pdf]

Supplemental Online Content

Cook D, Taneja S, Krewulak K, et al; Canadian Critical Care Trials Group and Canadian Clinical Research Network. Barriers, solutions, and opportunities associated with adapting critical care clinical trials in the pandemic. *JAMA Netw Open.* 2024;7(7):e2420458. doi:10.1001/jamanetworkopen.2024.20458

Supplement 1. eAppendix 1. Self-Administered Survey Instrument

eAppendix 2. Focus Group Interview Guide

This supplemental material has been provided by the authors to give readers additional information about their work.

© 2024 Cook D et al. *JAMA Network Open.*

eAppendix 1. Self-Administered Survey Instrument

1. **Please indicate the overall importance of the following barriers on a scale of 1-5 with respect to completing your RCT. Please consider the first 2 years of the pandemic period overall from March 11, 2020 to March 11, 2022 when responding.**

Possible Barrier	Irrelevant (1)	Not Important (2)	Neutral (3)	Important (4)	Very Important (5)
Hesitancy about research among the lay public, families and patients					
Hesitancy about research among colleagues, health professionals					
Less family presence in the ICU					
Insufficient remote access to medical charts for Research Coordinators to work from home at the start of the pandemic					
Periodic need for Research Coordinators to work from home for family reasons throughout the pandemic					
Local Research Coordinator(s) deployed to the bedside					
Local Research Pharmacy support limited due to clinical load or COVID research priorities					
Local decisions/directives to pause all clinical research					
Local decisions/directives to focus on COVID-19 specific research					
Large number of COVID-related RCTs					
Uncertainty about how to handle previously enrolled patients in your RCT when the pandemic was declared on March 11					
Delayed ethics approval for new centers participating in your RCT					
Delayed contract execution for new centers participating in your RCT					
Delayed site initiation visits for new centers participating in your RCT					
Re-establishing momentum for your RCT as the pandemic wanes					
Need for scientific protocol amendments for your RCT					
Need for protocol implementation amendments for your RCT					
Ongoing fixed costs and need for additional funding for your RCT					
Remote rather than in-person connection with collaborators during the pandemic					
The effect of infection precautions on your RCT procedures (devices, aerosolizing procedures, etc,)					
Other (please specify:_________)					
Other (please specify:_________)					

© 2024 Cook D et al. *JAMA Network Open.*

Other (please specify:_________)					

2. **Please share the 5 most helpful <u>solutions</u> you developed to complete your RCT during the pandemic.**
   Examples of strategies: seeking REB approval for telephone consent encounter, allowance of faxed signatures, incorporating the deferred consent model (e.g., consent to continue).

3. **Which barrier and associated solution do you believe had the <u>biggest positive impact</u> toward completion of your RCT?**
   Please specify:

   Barrier:____
   Solution:____
   Positive Impact:____

4. **Please share 5 new opportunities encountered when working toward completing your RCT specifically.**

   Opportunities/positive consequences specific to your RCT (e.g., ICU RC expertise was leveraged for pandemic trials outside ICU which ensured staff retention; principles were developed to guide non-COVID specific research during the pandemic; process enhancements for single-center multi-study management were developed; additional funding opportunities became available; sub studies and embedded SWATs ['study within a trial'] were developed).

5. **Please share 5 <u>new opportunities</u> encountered when working toward completing <u>your RCT (about research in general).</u>**

6. **Please share 5 unintended positive consequences encountered when working toward completing your RCT (either about your RCT, or about research in general).**

   Opportunities/positive consequences to research in general (e.g., increased public awareness of ICUs, and of research; unmasking possible contract efficiencies; benefits of platform trials; community hospital participation in COVID RCTs and continued RCT engagement; the pandemic allowed a longer lead time to start other non-COVID RCTs)

© 2024 Cook D et al. *JAMA Network Open.*

7. **Please share 5 <u>suggestions</u> for investigators conducting RCTs in the future based on your experiences conducting an RCT during the pandemic (e.g., your main lessons learned).**

   Examples of lessons learned: virtual/hybrid cost-effective eco-friendly site initiation visits; remote monitoring.

8. **Upon reflection, what would you say is your most important suggestion for future trialists?**

9. **For future trialists, to what extent do you think your most important suggestion might be relevant?** (Please circle your response).

Small Extent(1) — Limited Extent(2) — Fair Extent(3) — Moderate Extent(4) — Large Extent(5)

Respondent Characteristics

1. Please identify your role in the RCT:
   ☐ Principal Investigator ☐ Project Coordinator
2. Do you identify as:
   ☐ Woman ☐ Man ☐ Non-binary gender ☐ I prefer not to answer
3. Do you identify as:
   ☐ Early-career (<5 years) ☐ Mid-career (5-15 years) ☐ Senior-career (>15 years)
4. Date of Survey Completion: ☐ ☐ / ☐ ☐/ ☐ ☐ (DD/MM/YY)

RCT Characteristics

1. Name of your RCT: ____
2. Clinicaltrials.gov or other RCT identifier: ____________________________
3. Population for your RCT (check all that apply): ☐ Adult ☐ Pediatric
4. Biological specimens incorporated into your protocol: ☐ Yes ☐ No
5. Date when the first patient was enrolled in your RCT: ☐ ☐ / ☐ ☐/ ☐ ☐ DD/MM/YY
6. Total projected sample size: ☐ ☐ ☐ ☐ Patients
7. Current status of your RCT:
   ☐ Projected final enrolment ☐☐/☐☐/☐☐ DD/MM/YY
   ☐ Final enrolment completed ☐☐/☐☐/☐☐ DD/MM/YY
8. Patient enrolment in your RCT?

Enrolment as of March 11th 2020	Enrolment now (or at RCT completion)
☐ ☐ ☐ ☐ Patients	☐ ☐ ☐ ☐ Patients

9. Sites participating in your RCT?

Enrolment as of March 11th 2020	Enrolment now (or at RCT completion)
☐ ☐ Sites	☐ ☐ Sites

10. Original pre-pandemic funding secured for your RCT:
    ☐ CIHR (please specify: ____________) ☐ Other peer-review agencies (please specify: ___)
    ☐ University (please specify: ________) ☐ Hospital (please specify: ___________________)

© 2024 Cook D et al. *JAMA Network Open.*

☐ Other (please specify e.g., Foundation____________________)

11. Did you apply for additional funding to complete your RCT on account of the pandemic:
    ☐ No
    ☐ CIHR (please specify: ____________) ☐ Other peer-review agencies (please specify: ___)
    ☐ University (please specify: ________) ☐ Hospital (please specify: ___________________)
    ☐ Other (please specify: e.g., Foundation____________________)
    ☐ Other COVID-specific funds (please specify: e.g., Accelerated Clinical Trials______________)

Thank you for your interest in this study!

© 2024 Cook D et al. *JAMA Network Open.*

eAppendix 2. Focus Group Interview Guide

Thank you for joining this focus group as a Project Coordinator or Principal Investigator for a CCCTG-related randomized trial that was ongoing when the pandemic was declared. Your participation today will help us further understand some points that were raised in the survey component of this study.

We identified three areas that we believe would benefit from further elaboration.

1. Which organizational components of the research enterprise do you believe will facilitate the timely launch and the timely completion of future RCTs in the ICU setting?

Prompts guided by responses….

2. How do you view the promise and the perils of technology for future RCTs in the ICU setting?
    a. For patients/families?

    Prompts guided by responses….

    b. For research teams?

Prompts guided by responses….

3. To be agile and respond to evolving circumstances such as a pandemic, what contingency plans do you believe should be in place in future RCTs in the ICU setting?

Prompts guided by responses….

We value your thoughts. Are there any other comments you'd like to share?

Thank you for your time and sharing your perspectives.
