# Supplementary material for: Barriers, Solutions, and Opportunities for Adapting Critical Care Clinical Trials in the COVID-19 Pandemic
Source: JAMA Netw Open. 2024 Jul 12;7(7):e2420458. doi: 10.1001/jamanetworkopen.2024.20458 (PMC11245722; doi:10.1001/jamanetworkopen.2024.20458)
Supplement: Supplement 2. — Data Sharing Statement [file jamanetwopen-e2420458-s002.pdf]

Data Sharing Statement

Cook. Barriers, Solutions, and Opportunities Associated with Adapting Critical Care Clinical Trials in the Pandemic. *JAMA Netw Open*. Published July 05, 2024.
doi:10.1001/jamanetworkopen.2024.20458

Data
Data available: No

Additional Information
Explanation for why data not available: Participants in this mixed-methods study were not asked to give informed consent to disseminate results of their focus group transcripts or self-reported surveys, and we do not have IRB approval for full data sharing. Data sharing for summary findings is possible.
